# Anticorrelations between Active Brain Regions: An Agent-Based Model Simulation Study

**DOI:** 10.1155/2018/6815040

**Published:** 2018-03-19

**Authors:** Fabrizio Parente, Alfredo Colosimo

**Affiliations:** Deparment of Anatomy, Histology, Forensic Medicine and Orthopedics, Sapienza University of Rome, Rome, Italy

## Abstract

Anticorrelations among brain areas observed in fMRI acquisitions under resting state are not endowed with a well-defined set of characters. Some evidence points to a possible physiological role for them, and simulation models showed that it is appropriate to explore such an issue. A large-scale brain representation was considered, implementing an agent-based brain-inspired model (ABBM) incorporating the SER (susceptible-excited-refractory) cyclic mechanism of state change. The experimental data used for validation included 30 selected functional images of healthy controls from the 1000 Functional Connectomes Classic collection. To study how different fractions of positive and negative connectivities could modulate the model efficiency, the correlation coefficient was systematically used to check the goodness-of-fit of empirical data by simulations under different combinations of parameters. The results show that a small fraction of positive connectivity is necessary to match at best the empirical data. Similarly, a goodness-of-fit improvement was observed upon addition of negative links to an initial pattern of only-positive connections, indicating a significant information intrinsic to negative links. As a general conclusion, anticorrelations showed that it is crucial to improve the performance of our simulation and, since these cannot be assimilated to noise, should be always considered in order to refine any brain functional model.

## 1. Introduction

The not-well-defined nature of negative correlations stimulated several authors to study the persistence of significant negative correlations by means of fMRI-specific correction methods and to propose a possible physiological role for them [1–4]. In this regard, however, a clear mechanism about how negative interactions are related to the positive ones is not available as yet. A rewarding approach to the problem would be the simulation of brain activity, which opens the door to mechanistic models amenable to validation by empirical data.

Different models have been proposed [5] to approximate the collective activity of neurons such as the conductance-based biophysical model [6–8] or the FitzHugh-Nagumo model [9, 10], by the mean-field [11] or mass action [12] formalisms. fMRI produces data at a mesoscopic level while brain activities are inspected at a much larger scale than that of single neurons. This implies that we have to imagine how the behavior of single functional units, of major importance for the current understanding of brain's activities, may influence the observations at a higher hierarchical level [13].

In order to reproduce the brain resting state from fMRI acquisitions, the long-range myelinated fiber connections by diffusion imaging, or the folded cortical surface by high resolution imaging [14–17], have been used as a background for the interactions between brain areas. Such interactions have been simulated using the Kuramoto model [18], the Ising model [19], and some discrete-time dynamical models [20, 21]. In the last case [20, 21], a stochastic cellular automaton approach was used by two well-established brain computational models, the susceptible-excited-refractory (SER) [22] model and the FitzHugh-Nagumo model [9].

An alternative approach to the large-scale brain modeling is to simulate the brain activity using the functional connectivity map itself as a background. In such a context, Joyce et al. [23] realized an agent-based brain-inspired model (ABBM) using both positive and negative values of functional connectivity. In general, an agent-based model (ABM) includes a set of agents whose reciprocal interactions are defined by a set of rules depending upon the system at hand. These models can exhibit emergent behavior as described by Wolfram [24].

Here we develop a model using an ABM model and a biologically plausible SER model, which should account for both positive and negative interactions between large-scale brain areas. Different levels of functional connectivity in the background modulate the goodness-of-fit of simulations, and we focus, in particular, on the fraction of negative links to test their role in the organization of structured networks.

## 2. Materials and Methods

### 2.1. Data Collection

The sample is composed of 30 selected functional images of healthy controls from the Beijing Zang dataset (180 subject) in the 1000 Functional Connectomes Classic collection (http://fcon_1000.projects.nitrc.org/indi/retro/BeijingEnhanced.html). Resting data were obtained using a 3.0 T Siemens scanner at the Imaging Center for Brain Research, Beijing Normal University. For each subject, a total of 240 volumes of EPI images were obtained axially (repetition time, 2000 ms; echo time, 30 ms; slices, 33; thickness, 3 mm; gap, 0.6 mm; field of view, 200 × 200 mm^2^; resolution, 64 × 64; flip angle, 90°). For the anatomical images, a T1-weighted sagittal three-dimensional magnetization prepared rapid gradient echo (MPRAGE) sequence was acquired, covering the entire brain: 128 slices, TR = 2530 ms, TE = 3.39 ms, slice thickness = 1.33 mm, flip angle = 7°, inversion time = 1100 ms, FOV = 256 × 256 mm, and in-plane resolution = 256 × 192.

### 2.2. Data Preprocessing

The first 10 scans of each subject were removed, and the remaining functional images were analyzed according to the procedures fully described elsewhere [25]. The SPM8 (Statistical Parametric Mapping) (Wellcome Department of Cognitive Neurology, London, UK) toolbox and the Functional Connectivity (CONN) toolbox were used in the preprocessing of data on a MATLAB R2010b platform.

**Figure 1:**
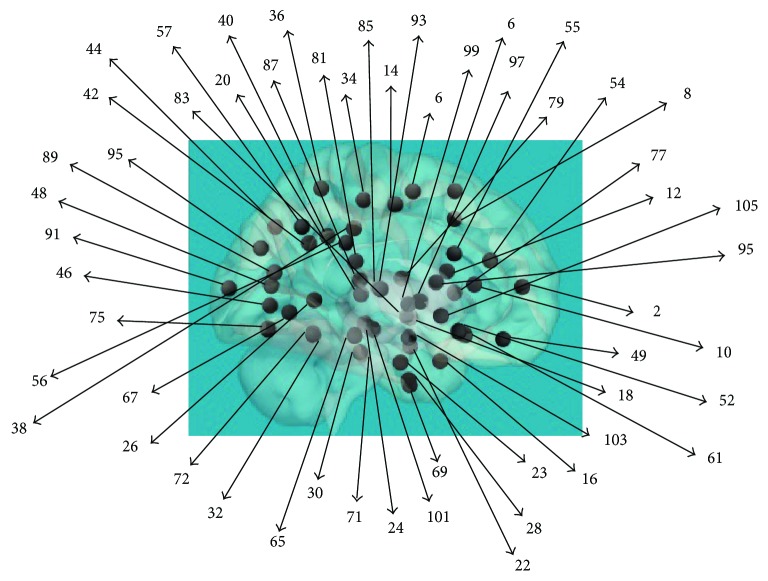
Brain parcellation. Location of the brain regions considered in the extraction of the BOLD signal and visible in a sagittal brain representation. For the complete list of the 105 regions considered in this work, taken from FSL Harvard-Oxford maximum likelihood cortical and subcortical atlas, see the Appendix.

**Figure 2:**
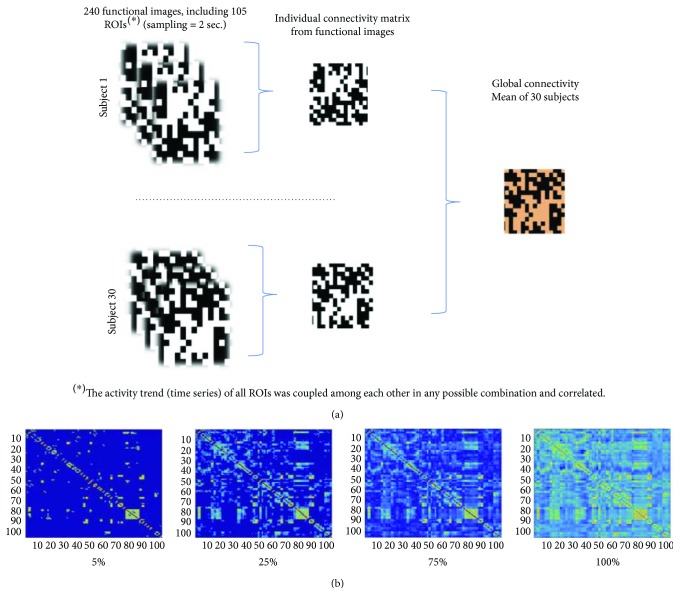
Working out the connectivity matrices. (a) Refers to point (1) of the procedure detailed in the text. The fractions in (b) concern the highest absolute correlation values of the threshold in the corresponding matrices (see point (2) in the text for details).

The images from each subject were divided into 105 ROIs without brainstem and cerebellum (see Figure 1) through the MRI Atlas of the Human Brain, Harvard Medical School [26], and from each ROI, the time series was extracted. An average correlation matrix for each subject was calculated for all possible couples of the 105 ROIs considering both correlation signs and was used as an (individual) connectivity matrix. Thus, the global, mean matrix to be used as a background for the brain simulation was reckoned according to the following overall procedure:

1. For each subject, the activation time series of 105 ROIs extracted from 240 functional images (see Data Collection) were coupled and correlated in all possible combinations, producing an individual connectivity matrix. Then, a global average concerning the whole group of subjects is obtained by averaging the 30 individual matrices, as schematized in Figure 2(a).
2. For both positive and negative interactions, in the above average matrix, a series of 20 binary and thresholded matrices are constructed, taking fractions of the highest absolute correlation values in the range from 0% to 100% at 5% steps: this represents the *network density* (cost). Thus, 20 binary matrices of increasing cost are derived, having an unbalanced amount of total positive and negative links (total positive correlations 70%, total negative correlations 30%). We call this type of threshold *absolute-values-proportional-threshold.* A graphical overview of the procedure is reported in Figure 2(b).
3. A further set of binary and thresholded matrices is calculated in order to distinguish the most significant correlation value for each sign: 15 matrices from the 0%–70% cost (maximum fraction of positive links), containing only positive values, and 7 matrices from the 0%–30% cost (maximum fraction of negative links), containing only negative values. Thus, we have different amounts of positive and negative correlations for the same fraction of total links. We call this type of threshold *signed-values-proportional-threshold*.
4. Finally, all the combinations of positive and negative matrices for different thresholds are joined, producing 7∗15 = 105 matrices having different amounts of positive and negative correlations.

### 2.3. Simulations by an ABBM Model

An agent-based approach was used in a large-scale brain network simulation able to account for the independent behavior of each brain region as well as for the interactions between different regions. Each node in the network represents, according to the susceptible-excited-refractory (SER) formalism [20, 21], a stylized biological neuron cycling in discrete time steps through the following three states: (S), a susceptible state in which the node can be excited with a transition probability called *sop*; (E), an excited state after which the node enters in a refractory state; and (R), a refractory state from which the node can be regenerated (S) stochastically with a recovery probability called *nep*.

The interactions among the nodes (agents) characterized by the (SER) states are defined through positive and negative links in a binary and thresholded matrix derived from empirical data and simulated through an agent-based brain-inspired model (ABBM) of the type suggested by Joyce [23].

**Figure 3:**
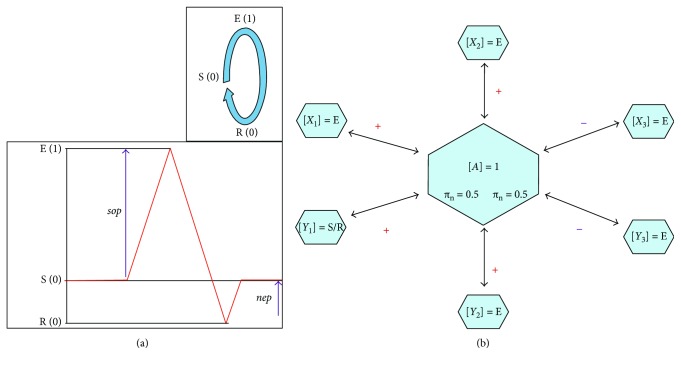
State balance of an agent (*A*) surrounded by six neighbors. (a) Activity levels of an agent in the SER (susceptible-excited-refractory) states: top and bottom pictures refer to a cycling scheme and to the classical action potential scheme, respectively. In parentheses are the 0/1 activity level of the state. *sop* and *nep* indicate the probability of getting the S → E and R → S state change, respectively (see the text for further details). (b) The state of the central node (*A*) in the next time step depends upon local (endogenous) and global (exogenous) factors. Three out of the four positively linked neighbors are active (1), so the average activity (3/4) exceeds the *φ*_p_ = 0.5 threshold. This is also the case for the both active (1) and negatively linked neighbors, since *φ*_n_ = 0.5 also.

In particular, each node is characterized by three variables (*φ*_s_, *φ*_p_, and *φ*_n_) and two parameters (*π*_p_ and *π*_n_) (see Figure 3), which are defined as follows.

- *φ*_s_ = 1 if the node is in the S (susceptible) state, namely, prone to change (otherwise, *φ*_s_ = 0).
- *φ*_p_ and *φ*_n_ are calculated from the average contribution of positive and negative neighbors, respectively; each neighbor contributes to the average if in the active (on) state.
- *π*_n_ and *π*_p_ are threshold parameters above which the average of negative and positive neighbors (*φ*_p_ and *φ*_n_) are set to 1 (otherwise, are set to 0).

Taking into account the previous variables, we characterized an agent by three binary variables (*φ*_s_, *φ*_p_, and *φ*_n_), namely, by one of 2^3^ possible combinations (111, 110, 101, 011, 100, 001, 010, 000). Simulations were carried out concurrently for all agents and for each step, and in contrast with Morris and Lecar [6], we designed some a priori rules to decide whether or not a brain region could become active at a given simulation step (Table 1).

Various combinations of the *sop*, *nep* (connectivity independent) and *π*_p_, *π*_n_ (connectivity dependent) couples of parameters have been checked in the above-described model in order to simulate at best the whole empirical, positive connectivity matrix by a given fraction of positive and negative links. In particular, if negative links are associated with noise, the simulation quality should decrease when their fractional amount increases and, inversely, increase in the opposite, symmetrical condition.

**Figure 4:**
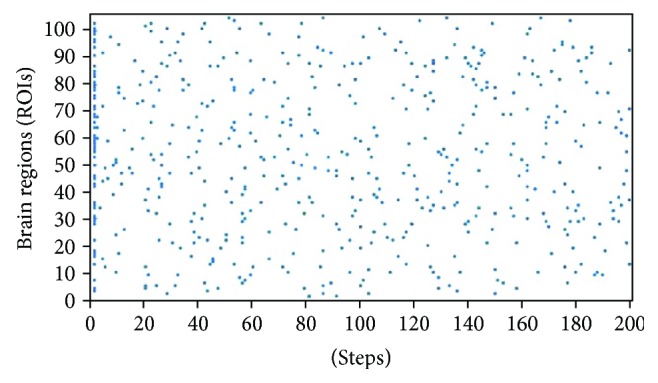
Example of a simulated time series. The time series corresponds to the condition included in Figure 5(b) (blue curve), namely, to the following parameter values: *sop* = 0.225, *nep* = 0.025, *π*_p_ = *π*_n_ = 0.1, and absolute-values-proportional-threshold = 100%. The spots indicate an excited state (E) for each of the 105 brain regions in each step of the simulation.

Simulations were repeated 100 times for each different combination of parameters, assigning to nodes a random series of 0 and 1 and a random SER state. Notice that in the case of the *π*_p_, *π*_n_ couple, the same value for each member of the couple was used. Each simulation included 200 time steps and produced a matrix of 105 columns (brain regions) and 200 rows (total time steps); see Figure 4. The Pearson correlation (*r*) carried out on the columns of such a matrix produced a 105 × 105 simulated connectivity matrix. The Pearson correlation between each of the 100 simulated matrices and the one derived from experimental data produced 100 correlations values for each combination of parameters which were averaged and the average value assigned to that parameter combination. It is worthy to underline that the Pearson correlation (*r*) was used throughout this work as an index of the agreement (goodness-of-fit) between simulations and empirical data.

The whole procedure included three series of simulations: The first two series aimed to optimize the parameter values; in the third series, the importance of different fractions of negative and positive connectivities in the reproduction of the positive connectivity itself was estimated. In particular, the following should be noted:

- In the first series of simulations, each of the 20 matrices characterized by an absolute-values-proportional-threshold (from 0% to 100% of absolute value threshold with 5% steps) was used as a background, as well as large variations of the other parameters (*sop* and *nep* = 0.25–0.50–0.75; *π*_p_/*π*_n_ from 0.1 to 1, step 0.1).
- The second series of simulations aimed to improve the parameter precision within the range identified in the previous set of simulations.
- Finally, the third series of simulations was carried out upon considering, within the 105 matrices characterized by any possible combination of 15 positive and 7 negative signed-values-proportional-thresholds, the one showing the best simulation performance, namely, the best reproduction of the original connectivity pattern.

The significance of the fitting performance was assessed as follows: in order to check the effect of positive and negative connectivities, 15 and 7 different fractions of positive and negative links, respectively, were used and subjected to a Friedman test. Then, a post hoc analysis using the ranks of the goodness-of-fit was performed by the Tukey-Kramer test.

## 3. Results

### 3.1. Exploring the Parameters' Space of the Brain Model

In the first exploratory phase of the model validation, the goodness-of-fit between empirical data and simulations, as monitored by the Pearson (*r*), was studied over a wide range of connectivity-independent (*sop*, *nep*) and connectivity-dependent (*π*_p_, *π*_n_) parameters, namely, 0.25–0.50–0.75 and from 0.1 to 1 at 0.1 steps, respectively.

**Figure 5:**
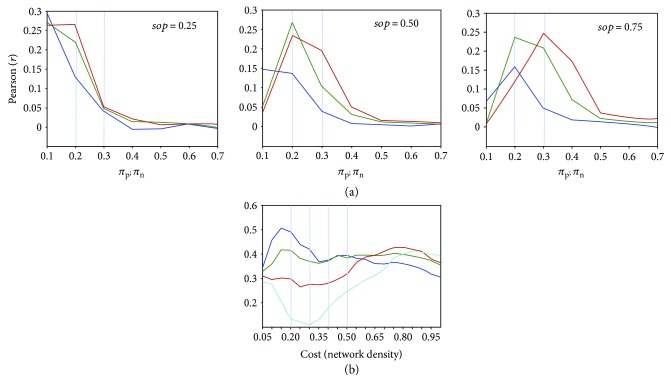
Fitting empirical data by the ABM model: dependence upon model's parameters. (a) Connectivity-dependent parameters (*π*_p_ and *π*_n_) on the x-axis. Blue, green, and red lines indicate, respectively, *nep* values of 0.25, 0.50, and 0.75. (b) Cost (network density) parameter on the x-axis; *sop* and *nep* fixed at 0.025 and 0.225, respectively. Blue, green, red, and light-blue lines indicate, respectively, 0.1, 0.075, 0.05, and 0.025 values of *π*_p_ and *π*_n_. Notice that a peak of the goodness-of-fit appears at *π*_p_, *π*_n_ = 0.1, in the lower range only of the network density. In all cases, the Pearson correlation (*r*) is used as a goodness-of-fit index.

In Figure 5(a), the *π*_p_ and *π*_n_ values associated with the goodness-of-fit peaks show a trend increasing with both *sop* and *nep* values. Since high *sop* and *nep* values point to an excitable system, endowed with high probability of spontaneous activation and low probability of resting in the refractory state, the fitting appears improved by a relatively conservative threshold for *π*_p_ and *π*_n_, namely, *π*_p_ and *π*_n_ = 0.1, under the condition of low excitability (*sop* and *nep* being equal to 0.25).

The above considerations suggest to focus on the lower range of parameters, namely, *sop* and *nep* from 0.025 to 0.25 (step = 0.025) and *π*_p_ and *π*_n_ from 0.025 to 0.1 (step = 0.025). Thus, the matching between simulation and empirical data could be improved by reaching the maximum value of 0.50 at the following connectivity-independent parameter values: *sop* = 0.025; *nep* = 0.175, 0.20, 0.225.

As shown in Figure 5(b), the highest goodness-of-fit is reached at *π*_p_ = *π*_n_ = 0.1 and using a small connectivity density (15%). At increasing *π*_p_ and *π*_n_ values, the trend changes gradually until at *π*_p_ = *π*_n_ = 0.1 an absolute minimum in the lower range of connectivity density can be observed, as well as a maximum in the higher range of connectivity density. Notice that *sop* and *nep* values are locked, respectively, at 0.025 and 0.225, and that changing the *nep* parameter does not alter the observed trends.

This behavior can be ascribed to the different amounts of positive and negative links using the absolute-values-proportional-threshold: The number of negative links is lower (almost nonsignificant for the lower level of general connectivity cost), and a more conservative threshold *π*_n_ would further decrease the associated information. Thus, with a more labile threshold of *π*_n_, more information from the negative connectivities can be extracted, which increases their modulation role. Due to the unbalanced distribution of positive and negative links, however, the simulation reaches a maximum value of goodness-of-fit only in the higher range of connectivity density (where a significant amount of negative connectivity is also increasing). At the same time, a lower threshold *π*_p_ can introduce random positive connections, decreasing the goodness-of-fit in the lower range of the connectivity density.

### 3.2. Modeling Positive and Negative Links

**Figure 6:**
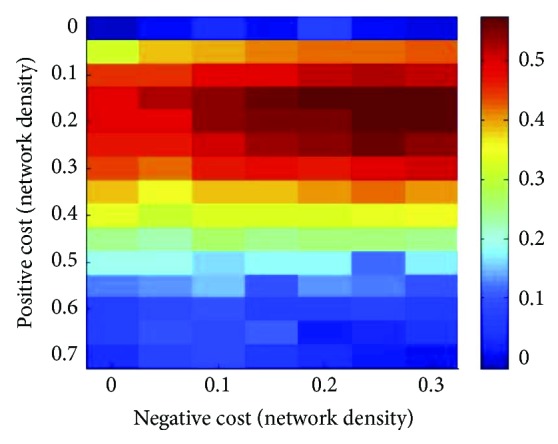
Fitting empirical data by combinations of positive and negative cost. The false-color scale visualizes the Pearson correlation between experiments and simulations obtained using the fractions of negative and positive links indicated in the horizontal and vertical axes, respectively.

In this phase, the task is to define the dependence of the fitting procedure on the relative amounts of positive and negative links, using the parameter values identified in the previous steps, namely, *sop* = 0.025, *nep* = 0.225, and *π*_p_ = *π*_n_ = 0.1. In Figure 6, the trend of correlation values at increasing positive connectivity fractions is characterized by a peak within the middle values of positive cost. Moreover, adding negative links at this stage further improves the fitting up to a maximum (0.57) at the higher values of negative network density.

**Figure 7:**
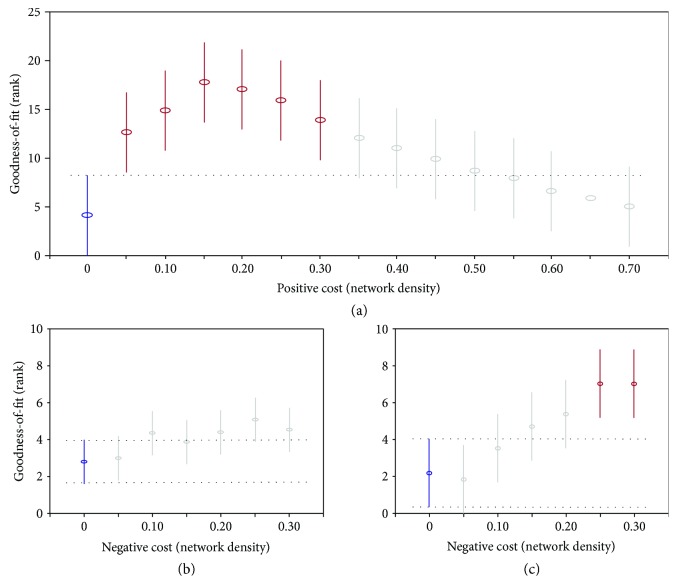
Post hoc analysis. Mean differences of the goodness-of-fit using an increasing amount of positive and negative links. (a) Goodness-of-fit as a function of positive links. (b) Goodness-of-fit as a function of negative links. (c) Goodness-of-fit as a function of negative links in the range of 5%–30% positive cost; a significant difference between the first mean value in blue (no negative links) is reached for the highest value (in red) of negative cost: 25%–30%.

A nonparametric statistical analysis (Friedman test) reported in Figure 7 confirms a significant effect (*p* < 0.0001, *χ*^2^ = 97.3, df = 1) of positive links on the fitting performance of the model. The effect of negative links, however, is not significant (*p* = 0.55, *χ*^2^ = 4.9, df = 6). The significant post hoc difference in the positive links is apparent in the range from 5% to 30% of positive network density (Figure 7(a)). The same nonparametric test for negative links in the range of higher values of goodness-of-fit is reported in Figure 7(c) where 6 different levels of positive cost (from 5% to 30%) are considered, while the levels of negative links remain 7. In contrast with previous results, under these conditions, a significant effect for the negative links (see Figure 7(c)*p* < 0.0001, *χ*^2^ = 37.1, df = 6) emerges. This indicates a possible interaction between different amounts of positive and negative links, so that only in the range of 5%–30% positive *cost* is there an increasing trend of goodness-of-fit upon addition of negative links (25%–30%). Under other conditions, only random fluctuations occur, probably caused by increasing variability levels.

### 3.3. Modeling Individual Variability

**Figure 8:**
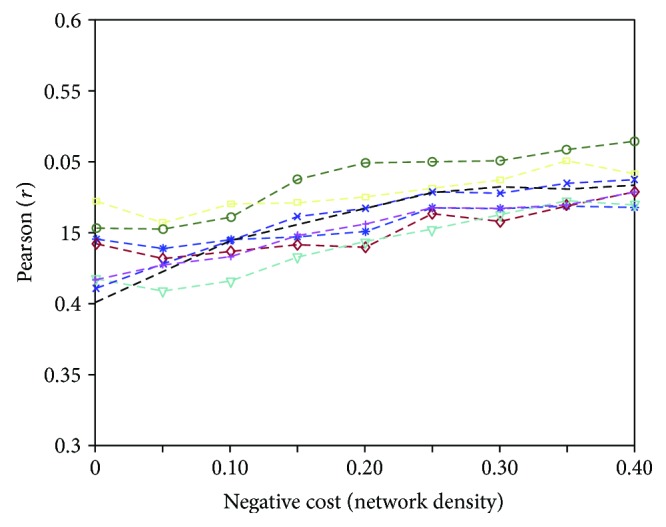
Modeling individual patterns. The goodness-of-fit values as a function of increasing amount of negative links (average of the fraction of positive links between 5% and 30%) concern 8 randomly chosen subjects. For the average values of the whole group of subjects, see Figure 7(c).

Given the noticeable level of individual variability in brain functional connectivity, the model has been individually applied on a small sample of subjects. For each of eight randomly chosen subjects, the simulations were repeated in the positive cost range indicated as significant by our previous work (positive cost: 5%–30%), and keeping the same values of the *sop*, *nep* and *π*_p_/*π*_n_ parameters. The results, shown in Figure 8, are in line with the previous observation of a small effect of anticorrelation variability in the model.

## 4. Discussion

### 4.1. General Issues about Our Brain Model

In this work, we propose a simple agent-based model able to simulate brain functional connectivity. Our results stress once again on how a set of simple rules between interacting agents can show a complex dynamics [24]. A peculiar feature of our work is the input used for the simulation: instead of the structural connectivity [14–17], we used the functional connectivity itself as a background and did that to underpin the role of a given amount of signed connectivity. In particular, we focused on the relative fraction of positive and negative links, to characterize the whole brain functions.

Our simulations exploit the appealing features of an ABBM-based strategy already used for the same purpose among several possible alternatives [23]. This approach showed different patterns of dynamics, but only some particular combinations of parameters produced nontrivial results [23] and, in addition, often lack coherent biological interpretation. We initially used some parameter values directly inspired to a biological system, and the results were unsatisfactory. Thus, we shifted to a SER model with the agents' dynamics defined by the *sop* and *nep* parameters. In this way, the brain regions show a stochastic oscillation in line with more realistic models [14, 15], and the connectivity represents a modulation among brain oscillating dynamics. As the first result of the adopted modeling strategy, the characterization of the system at hand was significantly improved.

### 4.2. Modeling Brain Activity Using Different Amounts of Positive and Negative Links

Different trends were found by our simulations depending upon the relative amount of positive and negative connectivities: In the former case (positive connectivities), the goodness-of-fit shows a peak at lower *cost* values, and a decreasing trend follows; in the latter (negative connectivities), the goodness-of-fit shows an increasing trend with a maximum at the maximal fraction of negative links.

As for positive connectivities, the statistical analysis showed clear differences between the random model (no connections among nodes, and all brain regions showing random oscillations) in the range between 5% and 30%. This result is in line with previous findings pointing to a small-world topology in that range [27]: In the same range, the brain positive networks show an efficient balance between the segregation-integration properties, and brain regions can be clustered in different subnetworks without losing the possible information transfer among each other [28]. As for negative links, the goodness-of-fit shows a trend different from that of the random model only if the positive links are in the range 5%–30%: otherwise, the trend is lost. In this frame, negative links showed importance in order to improve the fitting and prove their nonartifactual nature, while a higher density of positive links may indicate a significant noise source.

The results gathered by our model on single subjects are in agreement with those on the average matrix, indicating a good reproduction of individual variability. As a more general validation of our study, the same analysis carried out over another set of 30 randomly chosen individuals from the same database (Beijing Zang dataset, the 1000 Functional Connectomes Classic collection) produced pretty similar results (not shown).

An objective interpretation of our observations should take into account several factors: (1) More positive than negative modulations could be favoured by our model; (2) the anticorrelations have a more variable dynamics, more dependent on experimental conditions. From this point of view, such interactions are characteristic of the resting state itself and have a more local than global meaning; (3) our preprocessing method (aCompCorr [29]) used for the fMRI analysis could be not good enough to characterize negative networks. The first issue can be tested using different types of simulations in order to work out models for negative connections. In this regard, we would need a more accurate large-scale brain modeling able to account for this type of brain interaction. As for the second issue, different evidence is prone to assess the local versus global nature of anticorrelations. As a matter of fact, two evidence pointed out these different hypotheses: Gopinath et al. [30] found intracluster anticorrelations in several task-positive networks (TPNs) during a resting state, indicating a possible state-dependent activity. However, more recently [4], we found a low-connection probability between the most connected nodes using anticorrelated functional networks (the highly connected nodes tend to avoid connections among each other, indicating a global network organization).

About the last issue, however, there is no univocal consensus, and alternative methods have been proposed [2], among which the aCompCorr appeared as a most reliable one [1].

A direct comparison of aCompCorr with GSR [31], however, did not allow us to provide a final answer to the general problem, which remains, then, still open to further exploration.

## 5. Conclusion

All in all, the target of the present work was not to develop an alternative to the already used large-scale brain models but to underpin the importance of different connectivity types for the brain system. To this aim, we introduced a simple model able to fit empirical data, provided a method to identify the random (or noisy) functional connections, and found some evidence about the importance of anticorrelations for the optimal characterization of connectivity patterns.

It seems fair to conclude that anticorrelations (1) should be distinguished from noise and (2) may improve the characterization of positive connectivity and contribute to the refinement of the global brain functional system in fMRI acquisitions.

## Figures and Tables

**Table 1:** Transition rules adopted in the model.

*φ*_s_	*φ*_p_	*φ*_n_	State transition
0	0	0	E → R; R → S
0	1	1	E → R; R → S
0	0	1	E → R; R → S
0	1	0	E → R; R → S
1	0	0	S → E; S → S
1	1	1	S → E; S → S
1	0	1	S → S
1	1	0	S → E

The fourth column reports the type of transition at a given step (*i* → *i* + 1) depending upon the combinations of the *φ* values in columns 1–3.

## Appendix

### Anatomical Labels of Brain Regions

1. FP r (frontal pole right)
2. FP l (frontal pole left)
3. IC r (insular cortex right)
4. IC l (insular cortex left)
5. SFG r (superior frontal gyrus right)
6. SFG l (superior frontal gyrus left)
7. MidFG r (middle frontal gyrus right)
8. MidFG l (middle frontal gyrus left)
9. IFG tri r (inferior frontal gyrus, pars triangularis right)
10. IFG tri l (inferior frontal gyrus, pars triangularis left)
11. IFG oper r (inferior frontal gyrus, pars opercularis right)
12. IFG oper l (inferior frontal gyrus, pars opercularis left)
13. PreCG r (precentral gyrus right)
14. PreCG l (precentral gyrus left)
15. TP r (temporal pole right)
16. TP l (temporal pole left)
17. aSTG r (superior temporal gyrus, anterior division right)
18. aSTG l (superior temporal gyrus, anterior division left)
19. pSTG r (superior temporal gyrus, posterior division right)
20. pSTG l (superior temporal gyrus, posterior division left)
21. aMTG r (middle temporal gyrus, anterior division right)
22. aMTG l (middle temporal gyrus, anterior division left)
23. pMTG r (middle temporal gyrus, posterior division right)
24. pMTG l (middle temporal gyrus, posterior division left)
25. toMTG r (middle temporal gyrus, temporooccipital part right)
26. toMTG l (middle temporal gyrus, temporooccipital part left)
27. aITG r (inferior temporal gyrus, anterior division right)
28. aITG l (inferior temporal gyrus, anterior division left)
29. pITG r (inferior temporal gyrus, posterior division right)
30. pITG l (inferior temporal gyrus, posterior division left)
31. toITG r (inferior temporal gyrus, temporooccipital part right)
32. toITG l (inferior temporal gyrus, temporooccipital part left)
33. PostCG r (postcentral gyrus right)
34. PostCG l (postcentral gyrus left)
35. SPL r (superior parietal lobule right)
36. SPL l (superior parietal lobule left)
37. aSMG r (supramarginal gyrus, anterior division right)
38. aSMG l (supramarginal gyrus, anterior division left)
39. pSMG r (supramarginal gyrus, posterior division right)
40. pSMG l (supramarginal gyrus, posterior division left)
41. AG r (angular gyrus right)
42. AG l (angular gyrus left)
43. sLOC r (lateral occipital cortex, superior division right)
44. sLOC l (lateral occipital cortex, superior division left)
45. iLOC r (lateral occipital cortex, inferior division right)
46. iLOC l (lateral occipital cortex, inferior division left)
47. ICC r (intracalcarine cortex right)
48. ICC l (intracalcarine cortex left)
49. MedFC (frontal medial cortex)
50. SMA r (juxtapositional lobule cortex—formerly supplementary motor cortex right)
51. SMA L (juxtapositional lobule cortex—formerly supplementary motor cortex left)
52. SubCalC (subcallosal cortex)
53. PaCiG r (paracingulate gyrus right)
54. PaCiG l (paracingulate gyrus left)
55. AC (cingulate gyrus, anterior division)
56. PC (cingulate gyrus, posterior division)
57. Precuneus (precuneus cortex)
58. Cuneal r (cuneal cortex right)
59. Cuneal l (cuneal cortex left)
60. FOrb r (frontal orbital cortex right)
61. FOrb l (frontal orbital cortex left)
62. aPaHC r (parahippocampal gyrus, anterior division right)
63. aPaHC l (parahippocampal gyrus, anterior division left)
64. pPaHC r (parahippocampal gyrus, posterior division right)
65. pPaHC l (parahippocampal gyrus, posterior division left)
66. LG r (lingual gyrus right)
67. LG l (lingual gyrus left)
68. aTFusC r (temporal fusiform cortex, anterior division right)
69. aTFusC l (temporal fusiform cortex, anterior division left)
70. pTFusC r (temporal fusiform cortex, posterior division right)
71. pTFusC l (temporal fusiform cortex, posterior division left)
72. TOFusC r (temporal occipital fusiform cortex right)
73. TOFusC l (temporal occipital fusiform cortex left)
74. OFusG r (occipital fusiform gyrus right)
75. OFusG l (occipital fusiform gyrus left)
76. FO r (frontal operculum cortex right)
77. FO l (frontal operculum cortex left)
78. CO r (central opercular cortex right)
79. CO l (central opercular cortex left)
80. PO r (parietal operculum cortex right)
81. PO l (parietal operculum cortex left)
82. PP r (planum polare right)
83. PP l (planum polare left)
84. HG r (Heschl's gyrus right)
85. HG l (Heschl's gyrus left)
86. PT r (planum temporale right)
87. PT l (planum temporale left)
88. SCC r (supracalcarine cortex right)
89. SCC l (supracalcarine cortex left)
90. OP r (occipital pole right)
91. OP l (occipital pole left)
92. Thalamus r
93. Thalamus l
94. Caudate r
95. Caudate l
96. Putamen r
97. Putamen l
98. Pallidum r
99. Pallidum l
100. Hippocampus r
101. Hippocampus l
102. Amygdala r
103. Amygdala l
104. Accumbens r
105. Accumbens l
